# Revised Annotation and Characterization of Novel *Aedes albopictus* miRNAs and Their Potential Functions in Dengue Virus Infection

**DOI:** 10.3390/biology11101536

**Published:** 2022-10-19

**Authors:** Azali Azlan, Muhammad Amir Yunus, Mardani Abdul Halim, Ghows Azzam

**Affiliations:** 1 School of Biological Sciences, Universiti Sains Malaysia, Gelugor 11800, Pulau Pinang, Malaysia; 2 Infectomics Cluster, Advanced Medical & Dental Institute, Universiti Sains Malaysia, Kepala Batas 13200, Pulau Pinang, Malaysia; 3 Biotechnology Research Institute, Universiti Malaysia Sabah, Jalan UMS, Kota Kinabalu 88400, Sabah, Malaysia; 4 Malaysia Genome and Vaccine Institute, National Institutes of Biotechnology Malaysia, Jalan Bangi, Kajang 43000, Selangor, Malaysia

**Keywords:** *Aedes albopictus*, miRNA, small RNA-sequencing

## Abstract

**Simple Summary:**

*Aedes albopictus* (*Ae. albopictus*) is an important vector of the dengue virus. Genetics and molecular studies of virus infection in mosquito vectors are important to uncover the basic biology of the virus. It has been reported that miRNAs are important and possess functional roles in virus infection in *Ae. albopictus*. Here, we report a comprehensive catalog of miRNAs using the latest genome version of *Ae. albopictus*. We discovered a total of 72 novel mature miRNAs, 44 of which were differentially expressed in C6/36 cells infected with the dengue virus. Target prediction analysis revealed that the differentially expressed miRNAs were involved in lipid metabolism and protein processing in the endoplasmic reticulum. Results from this study provide a valuable resource for researchers to study miRNAs in this mosquito vector, especially in host–virus interactions.

**Abstract:**

The Asian tiger mosquito, *Ae. albopictus*, is a highly invasive species that transmits several arboviruses including dengue (DENV), Zika (ZIKV), and chikungunya (CHIKV). Although several studies have identified microRNAs (miRNAs) in *Ae. albopictus*, it is crucial to extend and improve current annotations with both the newly improved genome assembly and the increased number of small RNA-sequencing data. We combined our high-depth sequence data and 26 public datasets to re-annotate *Ae. albopictus* miRNAs and found a total of 72 novel mature miRNAs. We discovered that the expression of novel miRNAs was lower than known miRNAs. Furthermore, compared to known miRNAs, novel miRNAs are prone to expression in a stage-specific manner. Upon DENV infection, a total of 44 novel miRNAs were differentially expressed, and target prediction analysis revealed that miRNA-target genes were involved in lipid metabolism and protein processing in endoplasmic reticulum. Taken together, the miRNA annotation profile provided here is the most comprehensive to date. We believed that this would facilitate future research in understanding virus–host interactions, particularly in the role of miRNAs.

## 1. Introduction

*Aedes albopictus* (*Ae. albopictus*) is an important vector of arboviruses including dengue (DENV), Zika (ZIKV), and chikungunya (CHIKV) [1]. This mosquito is a robust and highly invasive species in both temperate and tropical regions of the world [2]. Due to its high adaptability, *Ae. albopictus* has spread rapidly and is considered a public threat throughout the world; hence, studying the fundamental biology of this mosquito is important for controlling its aggressive spread.

Previous studies in *Ae. albopictus* have shown the importance of non-coding RNAs (ncRNAs) in their development and virus infection [3,4,5,6,7]. Regulatory ncRNAs in metazoans include small RNAs, which consist of micro-RNAs (miRNAs), short-interfering RNAs (siRNAs), and PIWI-interacting RNAs (piRNAs). Particularly, miRNAs are 18–22 nucleotides (nts) in length and function in the regulation of gene expression by targeting messenger RNAs (mRNAs), resulting in the post-transcriptional silencing of the target genes [8,9]. Furthermore, miRNAs have been shown to play important roles in the development, growth, and infection of animals [10,11,12]. For instance, through a next-generation sequencing approach, multiple studies have reported that the expression profiles of *Ae. albopictus* miRNAs were altered at different developmental stages, during virus infection, and upon diapause conditions [3,6,13,14,15]. These studies suggest the involvement of miRNAs in the regulation of *Ae. albopictus* development and virus infection.

High-quality genomes coupled with high-depth RNA-sequencing data are important for accurate and comprehensive annotation of coding and non-coding genes. The alignment of small RNA reads to the reference genome is an essential step to annotate miRNAs in a species. Several studies used the genomes of closely related species as a proxy reference to facilitate the miRNA expression analysis [6,13,16]. For example, previous studies by Su et al. 2017 and Su et al. 2019 used *Ae. aegypti* miRNAs in miRBase (http://www.mirbase.org/, accessed on 25 Augest 2022) to evaluate the expression of *Ae. albopictus* miRNAs in the midgut when exposed to (DENV) infection [6,13]. Both studies reported novel *Ae. albopictus* miRNAs without mapping small RNA reads against the *Ae. albopictus* genome. Although this approach does not greatly affect the differential expression analysis of highly abundant miRNAs in non-model organisms [17], the level of error in annotation is unknown. By using the genomes of closely related species to discover miRNAs of another organism, we limit ourselves to only miRNAs that are conserved by both species. miRNA target prediction software, such as miRDeep2 [18], will excise potential precursors from the genome of closely related species based on the alignment of small RNA reads. Therefore, the predicted precursor sequences, which are derived from the reference genome of closely related species, may or may not be the true precursors of the newly identified miRNAs.

In this study, we aim to investigate miRNA expression upon DENV1 infection in *Ae. Albopictus* C6/36 cells. Before identifying the list of differentially expressed miRNAs upon infection, we first re-annotated miRNAs in *Ae. albopictus* using the updated genome assembly. The most recent and updated genome assembly of *Ae. Albopictus* was released in 2019 (AalbF2) using PacBio technology [19]. We decided to use this genome version for miRNA annotation. To gain a comprehensive annotation catalog, we combined our C6/36 cell-derived sequencing libraries with 26 publicly available datasets. These datasets encompassed multiple stages of *Ae. albopictus* development including embryo, larvae, pupae, adult males, sugar-fed/blood-fed females, diapause, and non-diapause pharate larvae. Finally, we predicted target sites of differentially expressed miRNAs to gain insights into their regulatory roles in *Ae. albopictus* during virus infection.

## 2. Materials and Methods

### 2.1. Cell Culture and Virus

*Ae. albopictus* C6/36 cells were obtained from American Type Culture Collection (ATCC: CRL-1660) and were cultured in Leibovitz’s L-15 medium (Gibco, Waltham, MA, USA, 41300039), supplemented with 10% Fetal Bovine Serum (FBS, Gibco, 10270) and 10% Tryptose Phosphate Broth (TPB) solution (Sigma, St. Louis, MO, USA, T9157). The C6/36 cells were incubated at 25 °C without CO_2_. BHK-21 cells (ATCC: CCL-10) were cultured at 37 °C in Dulbecco’s modified Eagle Medium (DMEM, Gibco, 11995065), supplemented with 10% FBS (Gibco, 10270) and 5% CO_2_. Dengue virus serotype 1 (Hawaiian strain) was propagated in C6/36 cells and titered using BHK-21 cells. Determination of DENV1 titer was carried out using 50% tissue culture infectious dose-cytopathic effect (TCID50-CPE) as previously described [5,20,21]. DENV1 (Hawaiian strain) used in this study was a gift from David Perera, University Malaysia Sarawak.

### 2.2. Virus Infection, RNA Extraction and Sequencing

C6/36 cells were infected with DENV1 at multiplicity of infection (MOI) of 0.25. Three days after infection, RNA extraction was carried out using miRNeasy Mini Kit 50 (Qiagen, Hilden, Germany, 217004) according to the manufacturer’s protocol. Total RNA was then subjected to next-generation sequencing. The RNA-sequencing libraries were prepared using standard Illumina protocols and sequenced using the HiSeq-SE50 platform, generating paired-end reads of 50 bp in size.

### 2.3. Verification of DENV1 Infection in C6/36 Cells

To verify the presence of DENV1 infection in C6/36 cells, the total RNA of both uninfected and DENV1-infected samples was subjected to cDNA synthesis using the Tetro cDNA synthesis kit (Bioline, London, UK, BIO-65042). DENV1-specific primer was used in cDNA synthesis. The polymerase chain reaction (PCR) assay was performed using DENV1-specific primers [22].

### 2.4. miRNA Identification

Publicly available small RNA-seq datasets were downloaded from NCBI Sequence Reads Archive (SRA). Prior to downstream analyses, small RNA-seq adapters were clipped using Trimmomatic version 0.38 [23] and low-quality reads were removed. Reads of 18–32 bp size were retained for downstream analysis.

To identify putatively novel miRNAs, clean reads from all small RNA-seq used in this study were pooled and aligned to the *Ae. albopictus* genome (AalbF2, NCBI) using miRDeep2 [18]. We used miRNAs predicted by Palatini et al. 2020 as known miRNAs [19]. For known miRNA precursors, we used all precursor miRNAs within the arthropod family that were retrieved from miRBase2.1. Predicted precursors that had significant Randfold *p*-value (*p*-value < 0.05) were retained. Minimum miRDeep score was set to >4. Then, we retained predicted miRNAs that shared the same seed sequence as arthropod miRNAs. For miRNAs that did not share the same seed sequence, only predicted precursors that had at least 1000 reads of mature miRNAs or those having at least 10 reads of predicted star sequence were retained.

### 2.5. Differential Expression of miRNA

The quantifier module in miRDeep2 package was used to quantify miRNAs in uninfected and DENV1-infected libraries [18]. In brief, total small RNA reads of both uninfected and DENV1-infected libraries were aligned against precursor miRNAs, and the resulting number of mapped reads indicates the miRNA abundance within each sample. Raw counts generated from the quantifier module were subjected to further analysis in the R/Bioconductor environment using edgeR package [24].

### 2.6. miRNA Target Prediction and Functional Analysis

Target prediction was performed using miRANDA [25] and RNAhybrid [26] using 3′UTR extracted from the *Ae. Albopictus* annotation file. miRANDA predictions were performed using the following parameters: score cut-off 140, energy cut-off −20, gap open penalty −9, gap extension penalty −4, seed regions at positions 2–8. RNAhybrid predictions were performed using the following parameters: energy cut-off −20, *p*-value < 0.1, binding required in miRNA positions 2–7. Functional analysis was conducted using g:Profiler [27]. We used g:SCS threshold for multiple testing corrections.

## 3. Results and Discussion

### 3.1. RNA-Sequencing Libraries and Read Mapping

In this study, we generated high-depth small RNA-seq libraries which were derived from DENV1-infected and uninfected *Ae. albopictus* C6/36 cell lines. All reads generated in this study were deposited in Short Read Archive (SRA) with the accession number SRP193815. A total of 270 million cleaned reads of 18–32 bp in length were acquired from our pooled set of six C6/36 small RNA-seq libraries (Table 1). These 18–32 bp raw reads were aligned to the *Ae. albopictus* genome (AalbF2, NCBI) and the resulting alignment files were subjected to downstream miRNA prediction analysis. In general, more than 90% of clean reads aligned to the reference genome (Table 1).

**Figure 1:**
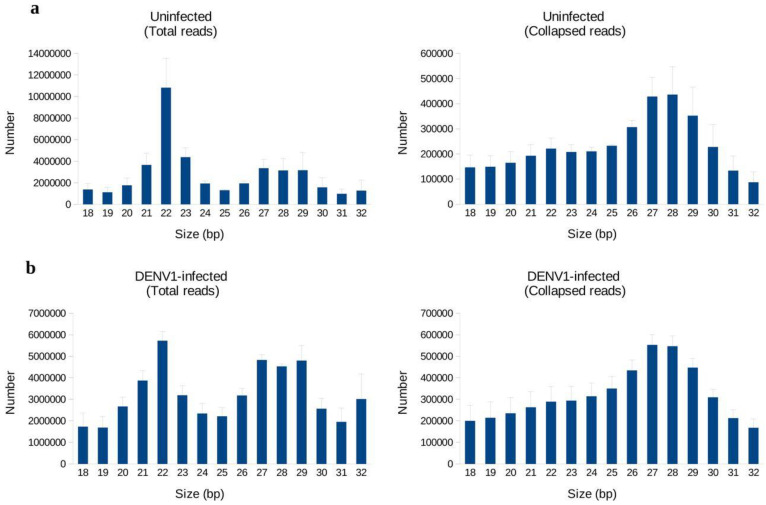
Length distribution of small RNAs in DENV1-infected and uninfected C6/36 cells. Size distribution of small RNA reads in uninfected samples (**a**) and DENV1-infected samples (**b**).

To visualize the heterogeneity of mapped reads in each library, the total number of small RNAs and their non-identical reads were plotted according to their size (Figure 1). The length distribution of total small RNAs revealed a major peak of 22 nts in size, but this peak collapsed when the total reads were converted into small RNA tags (Figure 1). The collapsibility of the peaks indicates the existence of miRNA in the libraries, since miRNAs are usually made up of relatively few identical sequences [28]. Unlike the 22 nt peak, peaks corresponding to longer small RNAs (24–32 nt) did not heavily collapse when converted into tags, suggesting the presence of a complex pool of small RNA populations [29].

### 3.2. Identification of Novel miRNAs in Ae. albopictus

To identify putatively novel miRNAs for *Ae. albopictus*, we combined reads from six libraries generated in this project with 26 public datasets (Supplemental Table S1), resulting in a total of approximately 350 million reads. The reads were then mapped to the genome (AalbF2, NCBI) and the resulting alignment file was fed into miRDeep2 for novel miRNA discovery. In our miRDeep2 analysis, we used miRNAs predicted from Palatini et al. 2020 as known *Ae. albopictus* miRNAs. We also defined other known miRNAs as miRNAs that have sequence conservations with miRNAs from other species, especially from the arthropod family curated from miRBase 2.1 (http://www.mirbase.org/, accessed on 25 August 2022). Most conserved miRNAs have been identified by previous studies [6,13,14,15,16,19,30].

A total of 72 novel mature miRNAs, which derived from 88 precursors, were identified. A complete list of both mature and precursor novel miRNAs identified in this study can be found in Supplemental Info S1. Palatini et al. 2020 reported a total of 121 mature miRNAs, which originated from 230 precursors from distinct loci in the genome of *Ae. albopictus* (AalBF2) [19]. We successfully confirmed the presence of miRNAs previously predicted by Palatini et al., 2020 in the AalbF2 genome version (Supplemental Info S1) [19]. Therefore, the total number of mature miRNAs in *Ae. Albopictus* is 193.

**Figure 2:**
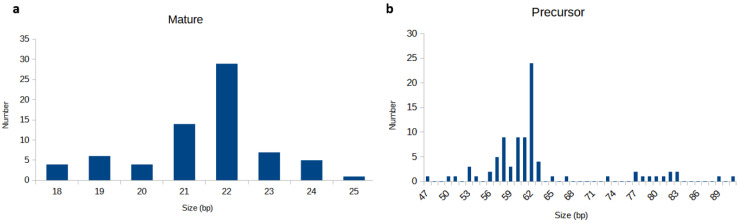
Size distribution of mature miRNA (**a**) and precursor miRNA (**b**).

The majority of mature *Ae. albopictus* miRNAs were found to be 22 bp in length, while in the case of precursor miRNAs, most of them ranged between 58 and 65 bp (Figure 2). *Ae. albopictus* miRNAs varied in terms of genomic loci—49.9% and 48.2% of them were located in the intergenic and intronic regions, while those remaining resided within the coding sequence (CDS).

We found that 29 of the novel miRNAs shared the same seed sequence with arthropod miRNAs curated from miRBase 2.1 (http://www.mirbase.org/, accessed on 25 August 2022) (Supplementary Info S1). Despite sharing the same seed sequence, these miRNAs were still considered novel because the predicted precursor sequences were not similar to known precursors. miRNAs interact with their target mRNA via base pairing of its “seed” sequence, which is usually in the position of 2–8 from the 5′ end of the mature miRNAs [31]. Hence, these 29 novel miRNAs may target the same genes as the known miRNAs with which they shared the same seed region.

The number of novel miRNAs detected in this study was higher compared to other *Ae. albopictus* studies [15,30]. For example, Gu et al. 2013 reported the discovery of 15 novel miRNAs, whereas Liu et al., 2015 and Batz et al., 2017 identified 5 and 10 novel miRNAs in *Ae. albopictus*, respectively. This finding is presumably due to the high sequencing depth and high percentage of mapped reads in every replicate of C6/36 small RNA libraries generated in this study. It was reported that libraries with a lower number of reads make the discovery of miRNAs, especially the less expressed, more challenging and somewhat limited [32].

We also discovered that multiple locations in the *Ae. albopictus* genome can give rise to the same mature and precursor miRNAs. A total of 118 distinct locations were found to be the genomic loci of 72 novel miRNAs, suggesting a gene duplication event. This was also true for *Ae. albopictus* protein-coding genes. The duplication of several members of gene families involved in insecticide-resistance, diapause, immunity, olfaction, and sex determination was observed in the *Ae. albopictus* genome, contributing to its large genome size [33].

From the standpoint of miRNA biogenesis, the generation of functional novel mature miRNAs requires the transcription of certain genomic loci that are capable of producing RNA whose secondary structures are recognizable by Drosha and Pasha (DGCR8 in vertebrates) in the nucleus, and Dicer and Loquacious in the cytoplasm [9,34,35,36,37,38]. Therefore, the birth of novel miRNA requires a relatively simple prerequisite, which is loci in the genome that evolve to produce RNAs that fold in such a way that miRNA protein machineries can identify and process them, resulting in fully functional mature miRNAs [39,40]. Due to the inherent property of RNA to form imperfect folding (the biogenesis of miRNAs requires an imperfect folding of RNA hairpins), the emergence of novel miRNAs, from the evolutionary viewpoint, is prone to being more feasible than the birth of new coding genes [40].

### 3.3. Expression of Novel miRNAs in Different Stages of Ae. albopictus Development

**Figure 3:**
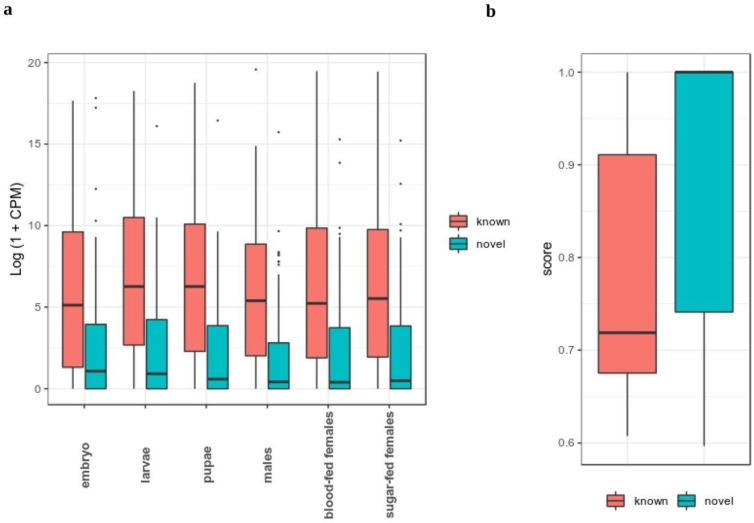
(**a**) Expression distribution of lncRNAs and protein-coding genes in *Ae. albopictus*. (**b**) Distribution of specificity score of lncRNAs and protein-coding genes in *Ae. albopictus*.

We then assessed the dynamics of novel miRNA expression throughout the *Ae. albopictus* development to have a better understanding of its distribution. We used the public dataset from Gu et al., 2013, which consists of six different stages, namely 0–24 h embryo, larvae, pupae, adult males, blood-fed females and sugar-fed females [30]. In general, the expression level of novel miRNAs was lower than known miRNAs across all six stages (Figure 3a). Next, we determined the specificity of novel miRNA expression across different developmental stages. We calculated the specificity score of each miRNA using the Jensen–Shannon (JS) score, which ranges from zero to one [41]. This specificity metric quantifies the similarity in expression value across developmental stages. miRNAs with a JS score of one indicate the extreme case in which they are only expressed in one specific stage, whereas those having a score close to zero are ubiquitously expressed in all stages with a relatively similar value of expression [41].

Here, we discovered that novel miRNAs were more specific in expression across different developmental stages compared to known miRNAs (Figure 3b). For instance, 23% of known miRNAs had a JS score of 1, while for novel miRNAs, 46% of them had a JS score of 1. Known miRNAs, on the other hand, have a more ubiquitous expression profile. This observation indicates that, unlike known miRNAs, novel miRNAs possess a narrower window of expression than known miRNAs. We speculate that novel miRNAs in *Ae. albopictus* possibly function as stage-specific tuners and regulators of genes involved in the control of developmental transition. Despite their lower level of expression, the specificity of novel miRNAs expression hinted to the possibility that they may execute distinct functions at specific developmental time-points.

### 3.4. Ae. albopictus Novel miRNAs Were Differentially Expressed upon DENV Infection

Previous studies have reported that *Ae. albopictus* miRNAs experienced changes in expression level upon DENV infection [3,6,13,14]. We asked if our newly annotated miRNAs were differentially expressed upon virus infection. We chose DENV serotype 1 (DENV1) in this study. DENV serotype 2 (DENV2) has been used in many genome-wide expression studies either in mammalian or mosquito hosts [42,43,44,45,46,47,48,49,50]. DENV2 has been the center of attention in studies involving DENV, despite the fact that other serotypes are similarly as important as serotype 2. Different serotypes of DENV display different clinical manifestations and levels of severity. A case study in Singapore reported that patients with a DENV1 infection may exhibit more severe illnesses compared to those infected with DENV2 [51]. Furthermore, different symptoms were observed between DENV1- and DENV2-infected patients. This study suggests that different serotypes of DENV can or may exhibit different clinical manifestations and levels of severity [51]. Therefore, studies involving different serotypes of DENV are important to provide insights into molecular mechanisms or virus pathogenesis.

**Figure 4:**
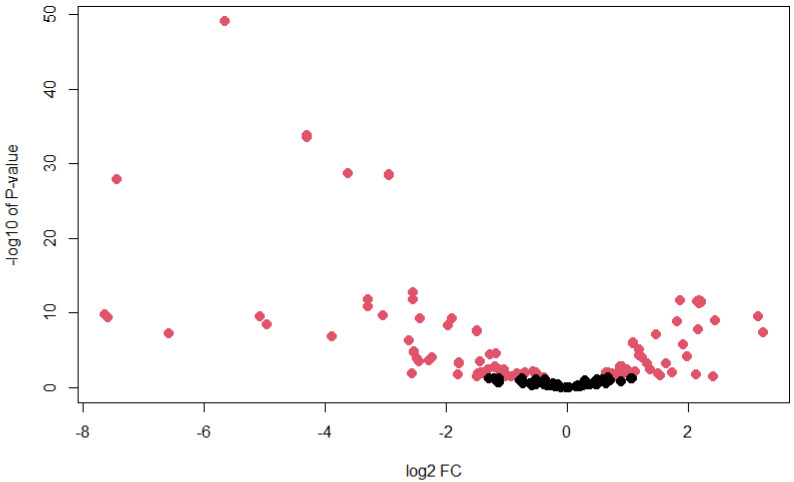
Volcano plot of differentially expressed miRNAs upon DENV1 infection. Each dot represents miRNAs (novel and known) with their corresponding log2 fold change and −log10 *p*-value. Red dots represent significant *p*-value (<0.05), while black dots represent insignificant *p*-value (>0.05).

In this study, we discovered that our newly annotated *Ae. albopictus* miRNAs in C6/36 cells were differentially expressed after 3 days post-infection with DENV1. A total of 143 miRNAs were found to be differentially expressed (*p*-value < 0.05), whereby 44 of them were novel miRNAs. A full list of differentially expressed miRNAs and their corresponding *p*-values can be found in Supplemental Table S2. To visualize the relationship between log fold change (FC) and *p*-value, a volcano plot was plotted (Figure 4). From the plot, we observed that value of log2 FC ranged between −8 and 4.

Avila-Bonilla et al., 2017 performed a small RNA-sequencing of C6/36 cells that were persistently infected with DENV2 to analyze changes in miRNA expression profile (Avila-Bonilla et al., 2017). Several differentially expressed miRNAs in C6/36 cells persistently infected with DENV2, such as miR-190, miR-970, miR-263a, miR-927, and miR-92b, were also found to be differentially expressed in our DENV1-infected C6/36 cells [14]. Therefore, we hypothesized that these miRNAs may be involved in host–DENV interaction in *Ae. albopictus*, regardless of the virus serotype. Meanwhile, other miRNAs may participate in the serotype-specific antiviral immunity in *Ae. albopictus*.

It was discovered that the overexpression of miR-927 in C6/36 cells resulted in a higher viral titer and viral genome copy number of DENV2. This result suggests that miR-927 may possess pro-viral activity in *Ae. Albopictus* cells [52]. Furthermore, the same group also showed that *filamin* (FLN), which has been associated with the regulation of the Toll pathway, is the direct target of miR-927 by a dual-luciferase gene reporter assay [52,53,54,55]. miR-927 was also found to be differentially expressed in this study, despite the serotype of DENV used being different. This further supports the idea that certain *Ae. albopictus* miRNAs are involved in host–DENV interaction, regardless of the virus serotype.

Next, we performed a miRNA target prediction to gain further insight into the potential downstream effects of regulation by differentially expressed novel miRNAs in C6/36 cells following DENV1 infection. Undeniably, the identification of miRNA target genes remains a complicated task. In silico analysis and computational methods are important in predicting potential miRNA target genes, despite the outcomes of such approaches varying from one algorithm to another [17]. To alleviate the certainty in the miRNA target prediction, we merged the outcomes from two miRNA target prediction tools, namely miRanda [25] and RNAhybrid [26]. miRNA binding sites derived from both software packages were deemed to be highly confident.

**Figure 5:**
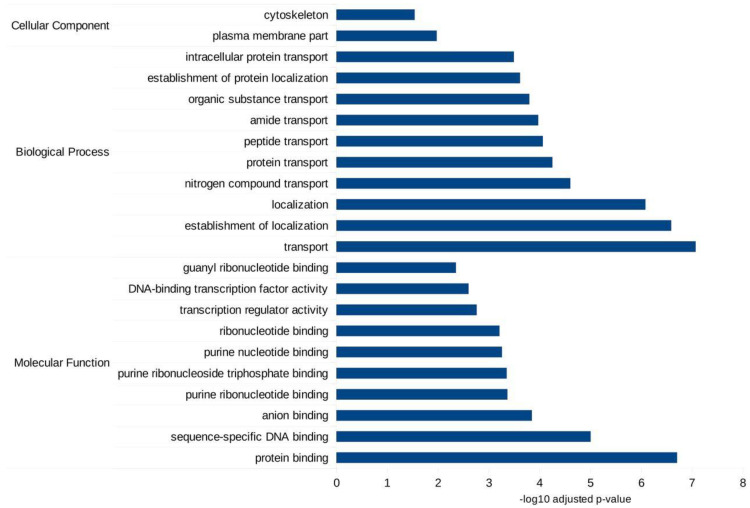
GO bar graphs of target genes of the differentially expressed miRNAs. Only the top 10 GO terms were plotted in the bar graphs.

Both miRANDA and RNAhybrid predicted a total of 10,867 genes as targets for differentially expressed miRNAs. Gene Ontology (GO) analyses using g:Profiler [27] showed that these target genes were involved in many types of biological processes, and molecular functions inside cells such as DNA and protein-binding, transport and localization (Figure 5). Meanwhile, KEGG pathway analysis revealed two important pathways: glycerophospholipid metabolism and protein processing in the endoplasmic reticulum (ER). A full list of GO and KEGG pathways from g:Profiler analysis can be found in Supplemental Table S4.

Previous studies have reported that among the cellular factors required for the life cycle of the enveloped virus, including DENV, are lipids [56,57,58]. DENV utilized a specific lipid, bis(monoacylglycerol)phosphate, which functions as a co-factor for viral fusion in late endosomes [58]. DENV entry into host cells is established via endocytosis and uses lipids as factors that trigger the fusion of viral particles into the endosome, hence delivering the viral genome into the cytosol [59,60,61]. One of the critical steps in the DENV life cycle is the translation of the viral genome into a single polypeptide chain by host ribosomes, and this process occurs in the rough endoplasmic reticulum [59,60]. Therefore, post-transcriptional regulation of proteins involved in lipid metabolism and protein processing in ER may be crucial in either facilitating or inhibiting virus replication.

The results presented in this study are exclusively based on immortalized C6/36 cells. One major limitation of cell line research is that it does not reflect the true nature of virus infection and pathogenesis in the whole organism. We expect that miRNA responses in *Ae. albopictus* adult mosquitoes are more complex than what we discovered here. However, data generated in this study are valuable for forthcoming explorations of miRNA functions in virus infection, especially in *Aedes* mosquitoes. Our work uncovers a part of the still-growing area of research in host–DENV interaction and offers insights into new research directions to pursue.

## 4. Conclusions

In summary, we provided a comprehensive genome-wide reannotation of *Ae. albopictus* miRNAs and their responses in DENV infection in C6/36 cells. Although our understanding of the functional roles of host miRNAs in DENV infection is somewhat limited, results generated in this study provide valuable resources for future research. Further research is required to fully understand the role of specific miRNAs in DENV infection and replication inside mosquito cells.

## Figures and Tables

**Table 1:** Mapping statistics of small RNA reads generated in this study.

Sample	Raw Reads	Clean Reads	18–32 bp Reads	Alignment Rate (%)
DENV replicate 1	78933646	76813221	51893885	95.73
DENV replicate 2	77299313	74393164	50852534	96.02
DENV replicate 3	55658966	54155383	42163819	95.29
Uninfected replicate 1	54757609	53082234	41476616	96.62
Uninfected replicate 2	52985076	51516847	41877287	95.78
Uninfected replicate 3	58901861	57008973	41997868	95.31

## Data Availability

All reads generated in this study are available in Short Read Archive (SRA) with the accession number of SRP193815.

## Supplementary Materials

The following supporting information can be downloaded at: https://www.mdpi.com/article/10.3390/biology11101536/s1, Supplemental Info S1: List of known and novel miRNAs in *Ae. albopictus* genome-wide; Supplemental Table S1: List of public datasets used in this study; Supplemental Table S2: List of differentially expressed miRNAs upon DENV1 infection in C6/36 cells; Supplemental Table S3: List of primers used in this study; Supplemental Table S4: List of GO terms of target genes of the differentially expressed miRNAs.
