# Supplementary material for: Revised Annotation and Characterization of Novel Aedes albopictus miRNAs and Their Potential Functions in Dengue Virus Infection
Source: Biology (Basel). 2022 Oct 19;11(10):1536. doi: 10.3390/biology11101536 (PMC9598099; doi:10.3390/biology11101536)
Supplement: Supplementary file 1 [file biology-11-01536-s001.zip › Supplementary.pdf]

Supplemental Table 1: List of public datasets used in this study

Accession number	Condition	Reference/Publication
SRR5168339	Mature oocytes diapause replicate 1	Batz et al., 2017
SRR5168338	Mature oocytes diapause replicate 2	
SRR5168337	Mature oocytes diapause replicate 3	
SRR5168336	Mature oocytes non-diapause replicate 1	
SRR5168335	Mature oocytes non-diapause replicate 2	
SRR5168334	Mature oocytes non-diapause replicate 3	
SRR5168333	Pharate larvae diapause replicate 1	
SRR5168332	Pharate larvae diapause replicate 2	
SRR5168331	Pharate larvae diapause replicate 3	
SRR5168330	Pharate larvae diapause replicate 4	
SRR5168329	Pharate larvae non-diapause replicate 1	
SRR5168328	Pharate larvae non-diapause replicate 2	
SRR5168327	Pharate larvae non-diapause replicate 3	
SRR5168326	Pharate larvae non-diapause replicate 4	
SRR609263	Sugar-fed females	Gu et al., 2013
SRR609265	Adult males	
SRR609264	Pupae	
SRR609260	Larvae	
SRR609261	Blood-fed females	
SRR609439	0-24 hours embryo	
SRR5251234	persistently infected DENV2 replicate 2	Avila-Bonilla et al., 2017
SRR5251235	persistently infected DENV2 replicate 1	
SRR5251236	acutely infected DENV2 replicate 2	
SRR5251237	acutely infected DENV2 replicate 1	
SRR5251238	not infected replicate 2	
SRR5251239	not infected replicate 1	

List of differentially expressed miRNAs upon DENV1 infection in C6/36 cells

miRNA	novel or known	logFC	PValue	FDR
aal-miR-306	known	-5.64933	7.59E-50	1.82E-47
aal-miR-308-1	known	-4.29278	1.54E-34	1.85E-32
aal-miR-308-2	known	-4.29279	2.69E-34	2.15E-32
aal-miR-12	known	-3.61887	2.23E-29	1.34E-27
aal-miR-7-1	known	-2.94713	3.08E-29	1.48E-27
aal-miR-7-2	known	-2.94713	3.91E-29	1.56E-27
aal_miR-novel_28	novel	-7.43836	1.12E-28	3.85E-27
aal-miR-278-1	known	-2.54711	1.65E-13	4.89E-12
aal-miR-278-2	known	-2.5471	1.83E-13	4.89E-12
aal-miR-263a-2	known	-3.28594	1.56E-12	3.48E-11
aal-miR-9c	known	-2.55391	1.64E-12	3.48E-11
aal-miR-2a-1	known	1.858379	1.89E-12	3.48E-11
aal-miR-2a-2	known	1.858379	1.97E-12	3.48E-11
aal-miR-2a-3	known	1.858379	2.03E-12	3.48E-11
aal_miR-novel_42	novel	2.173253	2.23E-12	3.56E-11
aal_miR-novel_43	novel	2.207557	3.31E-12	4.80E-11
aal_miR-novel_45	novel	2.153384	3.40E-12	4.80E-11
aal_miR-novel_46	novel	2.212679	3.73E-12	4.97E-11
aal_miR-novel_44	novel	2.185309	5.97E-12	7.54E-11
aal-miR-263a-1	known	-3.28413	1.47E-11	1.77E-10
aal_miR-novel_12	novel	-7.64208	1.74E-10	1.99E-09
aal_miR-novel_10	novel	-3.04648	2.16E-10	2.36E-09
aal-miR-new19-2	known	-5.07004	3.02E-10	3.07E-09
aal-miR-new19-1	known	-5.07006	3.07E-10	3.07E-09
aal_miR-novel_51	novel	3.161899	3.45E-10	3.31E-09
aal_miR-novel_21	novel	-7.58782	4.60E-10	4.24E-09
aal-miR-new6	known	-1.89608	5.75E-10	4.98E-09
aal-miR-9b	known	-2.43827	5.81E-10	4.98E-09
aal-miR-1	known	2.436746	1.14E-09	9.44E-09
aal-miR-275	known	1.814652	1.49E-09	1.19E-08
aal-miR-new25-1	known	-4.95382	3.63E-09	2.81E-08
aal-miR-190	known	-1.97162	5.02E-09	3.77E-08
aal_miR-novel_11	novel	2.166299	1.77E-08	1.29E-07
aal-miR-71-2	known	-1.48624	2.10E-08	1.48E-07
aal-miR-71-3	known	-1.48624	2.19E-08	1.50E-07
aal-miR-71-1	known	-1.48624	2.87E-08	1.91E-07
aal_miR-novel_60	novel	3.233838	3.98E-08	2.58E-07
aal_miR-novel_20	novel	-6.57609	6.60E-08	4.17E-07
aal-miR-317	known	1.469808	7.79E-08	4.79E-07
aal_miR-novel_59	novel	-3.88102	1.70E-07	1.02E-06
aal_miR-novel_57	novel	-2.61711	4.90E-07	2.87E-06
aal-miR-13-2	known	1.08101	1.05E-06	6.00E-06

aal-miR-13-1	known	1.08101	1.10E-06	6.12E-06
aal-miR-13-3	known	1.08101	1.12E-06	6.12E-06
aal_miR-novel_38	novel	1.911292	1.56E-06	8.30E-06
aal-miR-279	known	1.187138	7.42E-06	3.87E-05
aal-miR-new7-2	known	-2.52643	1.58E-05	8.06E-05
aal-miR-new7-1	known	-2.5254	1.81E-05	9.04E-05
aal-miR-277	known	-1.17493	2.94E-05	0.000144
aal-miR-988	known	-1.26949	4.20E-05	0.000202
aal-miR-996	known	1.190874	5.03E-05	0.000237
aal_miR-novel_66	novel	1.983803	7.39E-05	0.000341
aal_miR-novel_27	novel	-2.2348	9.16E-05	0.000415
aal_miR-novel_41	novel	1.229107	0.000103	0.000456
aal_miR-novel_30	novel	-2.47702	0.000114	0.000496
aal-miR-219	known	-2.28909	0.000269	0.001153
aal-miR-new15-2	known	-2.44913	0.000285	0.0012
aal-miR-new15-4	known	-2.44894	0.000294	0.001217
aal-miR-new15-1	known	-2.44865	0.000308	0.001237
aal_miR-novel_50	novel	-1.43719	0.000309	0.001237
aal-miR-new15-3	known	-2.44846	0.000317	0.001247
aal-miR-31-3	known	-1.78114	0.000461	0.001784
aal-miR-31-2	known	-1.78192	0.000507	0.001932
aal_miR-novel_37	novel	1.628254	0.000532	0.001989
aal-miR-31-1	known	-1.78241	0.000539	0.001989
aal_miR-novel_9	novel	1.313109	0.000579	0.002107
aal-miR-125-2	known	0.914715	0.001677	0.005948
aal-miR-125-1	known	0.914715	0.001685	0.005948
aal-miR-980a	known	-1.1986	0.001768	0.006113
aal-miR-980b	known	-1.1986	0.001789	0.006113
aal-miR-new1-3	known	0.866449	0.001808	0.006113
aal_miR-novel_52	novel	-1.18858	0.001925	0.006418
aal-miR-new1-2	known	0.86645	0.002064	0.006701
aal-miR-new1-1	known	0.86645	0.002066	0.006701
aal_miR-novel_24	novel	0.988072	0.003441	0.01101
aal_miR-novel_4	novel	-1.11726	0.003649	0.011523
aal_miR-novel_6	novel	-1.04177	0.003782	0.011787
aal_mIR-novel_34	novel	1.364922	0.004048	0.012454
aal_miR-novel_55	novel	-1.30419	0.00455	0.013822
aal-miR-989-2	known	-1.04807	0.006597	0.01979
aal-miR-989-1	known	-1.04812	0.006862	0.020331
aal-miR-375-1	known	1.122599	0.007026	0.020565
aal-miR-2945-1	known	-0.56069	0.00714	0.020645
aal-miR-2945-2	known	-0.56069	0.0074	0.021142
aal-miR-375-3	known	1.122694	0.007502	0.021182
aal-miR-375-2	known	1.122711	0.007591	0.021184

aal-let-7-2	known	0.656841	0.008538	0.023553
aal-let-7-1	known	0.656841	0.00888	0.024218
aal-miR-2b-1	known	0.672402	0.0093	0.024261
aal-miR-276b-3	known	-0.5121	0.009433	0.024261
aal-miR-2b-2	known	0.672402	0.009464	0.024261
aal_miR-novel_14	novel	1.727281	0.009497	0.024261
aal_miR-novel_22	novel	0.95694	0.009545	0.024261
aal-miR-276b-4	known	-0.5121	0.009577	0.024261
aal-miR-2b-3	known	0.672402	0.009603	0.024261
aal-miR-276b-2	known	-0.5121	0.009972	0.02493
aal-miR-2a-6	known	0.648379	0.010155	0.025104
aal-miR-2a-5	known	0.648379	0.010251	0.025104
aal-miR-276b-1	known	-0.5121	0.010498	0.025191
aal-miR-2a-4	known	0.648379	0.010553	0.025191
aal-miR-998-2	known	-0.69068	0.010619	0.025191
aal-miR-998-1	known	-0.69068	0.010706	0.025191
aal_miR-novel_62	novel	0.87235	0.010926	0.025408
aal-miR-276a-2	known	-0.51211	0.01101	0.025408
aal_miR-novel_53	novel	-1.42071	0.011156	0.0255
aal-miR-276a-1	known	-0.51211	0.011871	0.026878
aal_miR-novel_67	novel	1.502719	0.011993	0.0269
aal-miR-5-3	known	-1.39721	0.012466	0.027702
aal-miR-5-2	known	-1.39729	0.012627	0.027803
aal-miR-new13-2	known	-2.55633	0.012846	0.028029
aal-miR-5-1	known	-1.39752	0.013119	0.028366
aal-miR-new13-1	known	-2.5561	0.013556	0.029049
aal_miR-novel_7	novel	0.749149	0.013856	0.029429
aal-miR-981a	known	-1.47702	0.015255	0.031966
aal-miR-281	known	-0.83506	0.015317	0.031966
aal-miR-34	known	-0.76025	0.015938	0.032974
aal-miR-981b	known	-1.47524	0.01617	0.033169
aal-miR-10	known	0.808108	0.016625	0.033814
aal_miR-novel_17	novel	2.12215	0.017114	0.034515
aal_miR-novel_72	novel	-1.09539	0.0174	0.034607
aal-miR-184-1	known	-0.51807	0.017554	0.034607
aal-miR-184-2	known	-0.51807	0.017592	0.034607
aal_miR-novel_31	novel	-1.80226	0.02023	0.03937
aal_miR-novel_69	novel	-0.70668	0.020341	0.03937
aal-miR-92b-2	known	-0.86056	0.023939	0.045962
aal_miR-novel_15	novel	1.52657	0.025643	0.048844
aal-miR-92b-1	known	-0.86063	0.026395	0.04988
aal-miR-137-1	known	-1.01941	0.029299	0.054935
aal-miR-137-2	known	-1.01891	0.030693	0.056725
aal-miR-927-1	known	-1.49586	0.030726	0.056725

aal-miR-927-2	known	-1.49587	0.030964	0.056728
aal_miR-novel_32	novel	-0.93071	0.03257	0.058884
aal-miR-932	known	2.406846	0.032632	0.058884
aal-miR-970	known	0.623937	0.036032	0.064535
aal-miR-11-1	known	-0.46105	0.037656	0.066944
aal-miR-11-2	known	-0.46105	0.038698	0.068291
aal_miR-novel_16	novel	0.821419	0.039563	0.069307
aal_miR-novel_26	novel	0.640726	0.040222	0.069952
aal_miR-novel_23	novel	0.621534	0.043964	0.075801
aal-miR-285	known	0.877544	0.044217	0.075801
aal_miR-novel_25	novel	0.604102	0.047518	0.080422
aal-bantam	known	-0.37747	0.047583	0.080422

Supplemental Table 3: List of primers used in this study

Primer	Sequence
aal_novel_98	TGAAATAATACATATAGCATCC
aal_novel_86	CTTAACAGATTGAAAGCTGAAA
aal_novel_71	ACTATTGTAGTAGCGATAAGATG
aal_novel_62	TTGATACGCTTGATACTCCTCC
aal_novel_11	TCGGATAACAGCATTCGGTTGA
aal_miR-998-5p	TAGCACCATGAGATTCAGCTC
aal_miR-989-5p	GTGTGCTTTGTGACAATGAGAT

Supplemental Table 4: List of GO terms of target genes of the differentially expressed miRNAs

source	term name	term id	adjusted p-value	-log10 adjusted p-value
GO:Molecular Function	protein binding	GO:0005515	1.97E-07	6.70479174603375
GO:Molecular Function	sequence-specific DNA binding	GO:0043565	9.94889369300971E-06	5.00222520967977
GO:Molecular Function	anion binding	GO:0043168	0.000142492380265	3.84620835879342
GO:Molecular Function	purine ribonucleotide binding	GO:0032555	0.000430151352924	3.36637870680591
GO:Molecular Function	purine ribonucleoside triphosphate binding	GO:0035639	0.000443729811125	3.35288139307269
GO:Molecular Function	purine nucleotide binding	GO:0017076	0.000552793955253	3.2574367145752
GO:Molecular Function	ribonucleotide binding	GO:0032553	0.000615818043821	3.21054759016977
GO:Molecular Function	transcription regulator activity	GO:0140110	0.001738746781564	2.75976366088207
GO:Molecular Function	DNA-binding transcription factor activity	GO:0003700	0.002501260985401	2.60184099095407
GO:Molecular Function	guanyl ribonucleotide binding	GO:0032561	0.004447729803985	2.35186160366575
GO:Molecular Function	guanyl nucleotide binding	GO:0019001	0.005383072419859	2.26896977746956
GO:Molecular Function	enzyme binding	GO:0019899	0.006181084233802	2.20893533794413
GO:Molecular Function	purine ribonucleoside binding	GO:0032550	0.007851308256477	2.10505797112358
GO:Molecular Function	purine nucleoside binding	GO:0001883	0.007851308256477	2.10505797112358
GO:Molecular Function	GTP binding	GO:0005525	0.007851308256477	2.10505797112358
GO:Molecular Function	lipid binding	GO:0008289	0.008047719337836	2.09432717793362
GO:Molecular Function	kinase activity	GO:0016301	0.009089102570204	2.04147899555129
GO:Molecular Function	cytoskeletal protein binding	GO:0008092	0.009478441329184	2.02326307382112
GO:Molecular Function	nucleotide binding	GO:0000166	0.012114886893985	1.91668063613214
GO:Molecular Function	nucleoside phosphate binding	GO:1901265	0.012114886893985	1.91668063613214
GO:Molecular Function	ribonucleoside binding	GO:0032549	0.013557237634372	1.8678287914691
GO:Molecular Function	Ran GTPase binding	GO:0008536	0.013964751392081	1.85496679143511
GO:Molecular Function	nucleoside binding	GO:0001882	0.016175069942314	1.79115383273065
GO:Molecular Function	microtubule binding	GO:0008017	0.020763762324526	1.6826939509807
GO:Molecular Function	small molecule binding	GO:0036094	0.022758105400212	1.64286389554946

GO:Molecular Function	binding	GO:0005488	0.027749683362733	1.55674196802247
GO:Molecular Function	kinase binding	GO:0019900	0.044286231717747	1.35373127193499
GO:Molecular Function	tubulin binding	GO:0015631	0.048407913577793	1.31508363541692
GO:Molecular Function	translation initiation factor binding	GO:0031369	0.049399835895724	1.30627449378283
GO:Biological Process	transport	GO:0006810	8.51E-08	7.06994247853219
GO:Biological Process	establishment of localization	GO:0051234	2.58E-07	6.5878507411063
GO:Biological Process	localization	GO:0051179	8.26E-07	6.08307998293039
GO:Biological Process	nitrogen compound transport	GO:0071705	2.48419778217594E-05	4.6048138302796
GO:Biological Process	protein transport	GO:0015031	5.60274909074619E-05	4.2515988262107
GO:Biological Process	peptide transport	GO:0015833	8.62963420463158E-05	4.06400761289162
GO:Biological Process	amide transport	GO:0042886	0.000106695450036	3.97185410041043
GO:Biological Process	organic substance transport	GO:0071702	0.000160156005672	3.79545677113447
GO:Biological Process	establishment of protein localization	GO:0045184	0.000243286887315	3.61388129805698
GO:Biological Process	intracellular protein transport	GO:0006886	0.000321347482484	3.49302509811086
GO:Biological Process	regulation of cellular metabolic process	GO:0031323	0.001116734595904	2.95205002941786
GO:Biological Process	protein localization	GO:0008104	0.001137107508363	2.94419847279533
GO:Biological Process	regulation of macromolecule biosynthetic process	GO:0010556	0.001347068934507	2.8706101792509
GO:Biological Process	regulation of cellular biosynthetic process	GO:0031326	0.001506735691224	2.82196292413548
GO:Biological Process	regulation of biosynthetic process	GO:0009889	0.001506735691224	2.82196292413548
GO:Biological Process	regulation of RNA metabolic process	GO:0051252	0.001742407898429	2.75885016890351
GO:Biological Process	regulation of nitrogen compound metabolic process	GO:0051171	0.001944608380649	2.71116784690786
GO:Biological Process	regulation of metabolic process	GO:0019222	0.002114589841486	2.67477385847994
GO:Biological Process	regulation of nucleobase-containing compound metabolic process	GO:0019219	0.002183633703617	2.66082021113192
GO:Biological Process	regulation of gene expression	GO:0010468	0.002185617362111	2.66042586803554
GO:Biological Process	macromolecule localization	GO:0033036	0.002219267234713	2.65379039872639
GO:Biological Process	regulation of primary metabolic process	GO:0080090	0.002384849514927	2.62253901993566
GO:Biological Process	regulation of macromolecule metabolic process	GO:0060255	0.002566239357879	2.59070283862444
GO:Biological Process	establishment of localization in cell	GO:0051649	0.002743643477944	2.56167232358524
GO:Biological Process	phosphorylation	GO:0016310	0.003453481307897	2.4617428902874

GO:Biological Process	regulation of cellular macromolecule biosynthetic process	GO:2000112	0.003470951542013	2.45955144947559
GO:Biological Process	cellular macromolecule localization	GO:0070727	0.003481604556889	2.45822055789234
GO:Biological Process	regulation of RNA biosynthetic process	GO:2001141	0.004512863243424	2.34554782702192
GO:Biological Process	regulation of nucleic acid-templated transcription	GO:1903506	0.004512863243424	2.34554782702192
GO:Biological Process	cellular protein localization	GO:0034613	0.006185964408549	2.20859258307436
GO:Biological Process	biological regulation	GO:0065007	0.007230796704623	2.14081384857561
GO:Biological Process	cellular localization	GO:0051641	0.007643615822622	2.11670114923452
GO:Biological Process	phosphorus metabolic process	GO:0006793	0.007684300800866	2.11439564308164
GO:Biological Process	intracellular transport	GO:0046907	0.009199332664334	2.03624367599237
GO:Biological Process	regulation of transcription, DNA templated	GO:0006355	0.011367291301216	1.94434301051739
GO:Biological Process	phosphate-containing compound metabolic process	GO:0006796	0.012039107973232	1.91940569054121
GO:Biological Process	regulation of biological process	GO:0050789	0.012141316007587	1.91573423716046
GO:Cellular Component	plasma membrane part	GO:0044459	0.010635837486157	1.97322830723784
GO:Cellular Component	cytoskeleton	GO:0005856	0.0287728437121	1.54101721327682
KEGG	Glycerophospholipid metabolism	KEGG:00564	0.00078674336832	3.10416690918926
KEGG	Protein processing in endoplasmic reticulum	KEGG:04141	0.013746309762266	1.86181387383253
